# Influence of Clinical Parameters and Anticoagulation on Intraprocedural Cerebral Microembolic Signals during Pulmonary Vein Isolation

**DOI:** 10.1371/journal.pone.0157886

**Published:** 2016-06-21

**Authors:** Robert Larbig, Ralf Dittrich, Simon Kochhaeuser, Patrick Leitz, Fatih Guener, Catharina Korsukewitz, Dirk Dechering, Christian Pott, Kristina Wasmer, Jan Schmitges, Monika Kerckhoff, Lars Eckardt, Gerold Moennig

**Affiliations:** 1 Division of Electrophysiology, Department of Cardiovascular Medicine, University of Muenster, Albert-Schweitzer-Campus 1, Building A1, 48149, Muenster, Germany; 2 Department of Neurology, University of Muenster, Albert-Schweitzer-Campus 1, Building A1, 48149, Muenster, Germany; 3 Helios Klinikum Krefeld, Department of Urology, Lutherplatz 40, 47805, Krefeld, Germany; Klinikum Guetersloh, GERMANY

## Abstract

**Objective:**

We had the objective to determine the impact of clinical parameters and anticoagulation status on cerebral microembolic signals (MES) during pulmonary vein isolation (PVI) for atrial fibrillation (AF).

**Background:**

Thromboembolism and stroke are the most feared complications of PVI. MES can help to evaluate embolic burden. It is unknown whether clinical parameters have an impact on embolic risk during PVI.

**Methods:**

In this retrospective analysis we investigated the impact of clinical parameters, including the CHADS_2_- and CHA_2_DS_2_-VASc-score, pulmonary vein variants and echocardiographic parameters on MES rates in patients that underwent PVI using three different ablation approaches (radiofrequency ablation (iRF), pulmonary vein ablation catheter (PVAC) with deactivated electrode pair 1 or 5 (PVAC-red) or PVAC without deactivation (PVAC-all).

**Results:**

118 AF patients (61±12 years) were included between 2011 and 2013 (Median: 489 MES during PVI). Patients were more likely to have more MES (within 4^th^ quartile) with the PVAC-all approach (60.7% vs. 25.0% (iRF) vs. 14.3% (PVAC-red) respectively (p<0.001). Patients with oral anticoagulation (OAC) pre-ablation were more likely to have lower MES-counts (1^st^-3^rd^ quartile); (65.6% vs. 35.7%; p = 0.005). Additionally, patients with lower MES counts (1^st^-3^rd^ quartile) had significantly higher INR values than those in the 4^th^ quartile (1.78 vs. 1.09; p = 0.029). 2 patients developed a potentially thromboembolic event during the procedure.

**Conclusion:**

Clinical predictors of cerebral emboli and stroke do not correlate with cerebral embolic burden during PVI. Pre-ablation OAC and increased INR values correlate with decreased MES-rates. Therefore, it might be beneficial to perform PVI with pre-ablation anticoagulation even in low risk patients.

## Introduction

Catheter ablation of atrial fibrillation (AF) is a well-established treatment option for symptomatic patients and numbers of interventions are rising[1]. Various technical tools such as the "Pulmonary Vein Ablation Catheter" (PVAC^®^, Medtronic Inc., Minneapolis, Minnesota, USA) or cryo-balloon have been developed to facilitate pulmonary vein isolation (PVI) and decrease procedure times.

Concerns have been raised about an increased incidence of silent cerebral embolism using phased RF ablation device (PVAC^®^) in comparison with irrigated tip RF ablation or cryoablation[2]. Recently our group confirmed these findings in a prospective study which was able to detect an increased rate of microembolic signals (MES) analyzed by transcranial Doppler ultrasound as a surrogate marker of embolic activity in patients undergoing PVAC^®^ ablation compared to irrigated tip RF ablation[3]. We were also able to link the number of MES with a subtle, diffuse post-procedural impairment of neuropsychological function highlighting the need to reduce microemboli during ablation[4].

It is unknown whether patient-specific clinical parameters correlate with elevated embolic rates. The identification of such parameters might help to predict the thromboembolic burden in patients undergoing catheter ablation of atrial fibrillation and might have impact on intraprocedural management. Therefore, we performed a retrospective analysis of clinical, anatomical and procedural parameters on MES rate during PVI.

## Methods

### Study participants

This retrospective study was performed according to good clinical practice. Individual patients were not identified. The study cohort included 118 patients with confirmed diagnosis of atrial fibrillation that were referred to our center between 2011 and 2013. No patient was excluded. An individual written consent was obtained by every patient. The study was approved by the ethics committee of the University of Muenster. The PVIs were performed before the era of uninterrupted anticoagulation.

### Admission/Physical examination/Medical history/Preexisting drugs/Lab values/ECG

In all eligible patients, exact classification of atrial fibrillation into one of the three groups (paroxysmal, persistent and long standing persistent)[1] was performed. Upon admission all patients underwent a thorough physical examination by a trained physician. Also information about the clinical severity (EHRA/NYHA), medication and concomitant diseases were obtained and CHA_2_DS_2_-VASc-Scores were calculated as previously described[1]. Laboratory data were gathered from the university patient database and a 12–lead ECG was recorded on admission and before discharge (MAC 3500, GE Healthcare, Little Chalfont, UK).

### Computer Tomography of the atrial anatomy

Prior to the ablation procedure an imaging study with thorax contrast-enhanced multislice computed tomography (TCT; Somatom Definition, Siemens healthcare, Forchheim, Germany) was performed to assess the atrial anatomy and the presence or absence of pulmonary vein variants. Pulmonary vein variants were evaluated in the entire cohort and included left common outflow (LCO), left common trunk (LCT), right common outflow (RCO), right middle pulmonary vein (RMPV), right top pulmonary vein (RTPV), early branching (EB) as previously described[5]. In 6 patients no TCT was performed due to either abnormal renal or thyroid function.

### Transthoracic/transesophageal Echocardiography

All patients received a transesophageal echocardiography prior to the ablation procedure. All echocardiographic studies were obtained by experienced investigators using a Philips iE 33 echocardiography system (Philips, Amsterdam, Netherlands) in a transesophageal approach prior to the ablation and a transthoracic approach postoperatively. Left atrial diameter (edge to-edge method, parasternal view) and left ventricular ejection fraction (biplane Simpson method) were measured and the left atrial appendage was analyzed to exclude intracardial thrombus. Only complete datasets were included in the statistical analysis.

### Ablation procedure (PVAC-red, PVAC-all, iRF)

Subsequently patients were transferred to the catheter lab where PVI was either performed by iRF or by PVAC solely due to operators′ preference and experience. All ablation procdures were performed using local anaesthetics and mild sedation when necessary.

### ‘Conventional’ phased radiofrequency ablation (PVAC-all)

After gaining femoral venous access, a steerable decapolar catheter (Livewire; St. Jude Medical) was placed in the coronary sinus. A single transseptal puncture (TSP) was done using a non-steerable sheath (ARRIVE, Medtronic Inc., Minneapolis, USA) under fluoroscopic imaging and pressure monitoring. The long sheath was perfused with heparinized saline solution. Subsequently, a bodyweight adjusted heparin bolus was applied. The target activated clotting time (ACT) was 250–350 s with half-hourly controls and managed with additional heparin boluses if necessary. Thereafter, selective angiography of the PVs was performed with about 40 mL of nonionic contrast (Ultravist 370; Bayer, Leverkusen, Germany), in posterior-anterior and left anterior oblique projections. The 10 pole phased RF catheter (PVAC; Medtronic Inc.) was positioned at the ostium of each pulmonary vein (PV) in an over-the-wire technique (PV-Tracker; Medtronic Inc.) and ablation was done exclusively with a 4:1 bipolar/unipolar ratio and a maximum power of 8W for 1 min. Temperature limit was set to 60°C. After each ablation, conduction into the PV was checked and ablation was repeated at sites with remaining conduction. Electrode pairs without tissue contact or positioned at sites without conduction were deactivated at the operators’ discretion. This procedure was repeated until no remaining conduction was detected. Complete isolation of the PVs was confirmed during sinus rhythm and using differential pacing maneuvers. If AF persisted after ablation sinus rhythm was restored by electrical cardioversion.

### ‘Modified’ phased radiofrequency ablation (PVAC-red)

After first evidence of increased incidence of silent cerebral embolism due to electrical interference between electrodes 1 and 10 (n = 29 PVAC-all) we modified the PVAC approach: Materials, procedural techniques, and settings equal those used during ‘conventional’ phased RF ablation, while electrode pairs 1 or 5 were deactivated throughout the procedure.

### Irrigated tip radiofrequency ablation (iRF)

In contrast to the phased RF ablation groups, access to the left atrium (LA) was achieved by performing two separate transseptal punctures (TSP). A non-steerable sheath (Daig SL1; St. Jude Medical, St. Paul, Minnesota, USA) for advancing the diagnostic circular decapolar catheter (Inquiry Optima; St. Jude Medical), and a deflectable long sheath (Agilis; St. Jude Medical) for the ablation catheter were placed in the LA. Management of anticoagulation including ACT measurements and heparin dosage were identical with the phased RF groups. After selective, simultaneous angiography of the ipsilateral PVs and acquisition of the individual three-dimensional anatomy of the LA (Ensite NavX Velocity; St. Jude Medical, with image integration), antral circumferential RF ablation around ipsilateral PVs using a 4 mm open-tip irrigated catheter (IBI Therapy Coolpath Duo; St. Jude Medical) was performed. Maximum power was set to 30W, going selectively up to 40W if PVI could not be achieved, especially at the anterior ridge border of the lateral PVs. Temperature was limited to 43°C. Irrigation was adjusted manually between 17 and 30 mL/min. Electrical cardioversion was performed if the patient remained in AF after PVI.

### Microembolus detection and ACT-detection

The detection and evaluation procedure was performed according to international consensus recommendations[6]. The same TCD device (Doppler-Box; Compumedics DWL) was used for all studies and for the blinded off-line evaluation of the data. Both medial cerebral arteries were continuously insonated through the temporal window from the start of the procedure (groin puncture) until the end of the ablation procedure (withdraw through the interatrial septum) in all patients. A continuous MES registration of at least one medial cerebral artery throughout the ablation procedure could be achieved in all patients. The investigations were well tolerated by the subjects, without any side effect. Two different observers (F.G., P.L.) analyzed the tapes off-line. Analysis of MES comprised (i) listening to each signal and (ii) watching each signal on screen at highest speed. Ratings were made without using a decibel threshold. All signals not classified as artefacts but as MESs were counted in each patient. Single countable MESs were summed up to a total MES count. Activated coagulation time (ACT) was measured using a ITC Hemochron Signature Elite (Keller Medical Inc. Bad Soden, Germany). An ACT of 250–350 ms was regarded as sufficient.

### Statistical analyses

Statistical analyses were performed using SPSS 22 software (IBM, Armonk, USA). The results are given as mean ± standard deviation (SD) or median (1^st^, 3^rd^ quartile) in the case of non-normal distribution. Each variable was tested for normal distribution using the Shapiro-Wilk test. Differences between groups and subgroups were evaluated by Chi-Square-Test and Mann-Whitney-U test. The predictive value of clinical parameters and scores for a high number of MES (within the 4^th^ quartile) were evaluated by univariable, binary regression analysis. The three best predictors in univariable testing were also evaluated by multivariable regression. A p<0.05 was considered as statistically significant for all tests.

## Results

### Baseline characteristics

**Fig 1:**
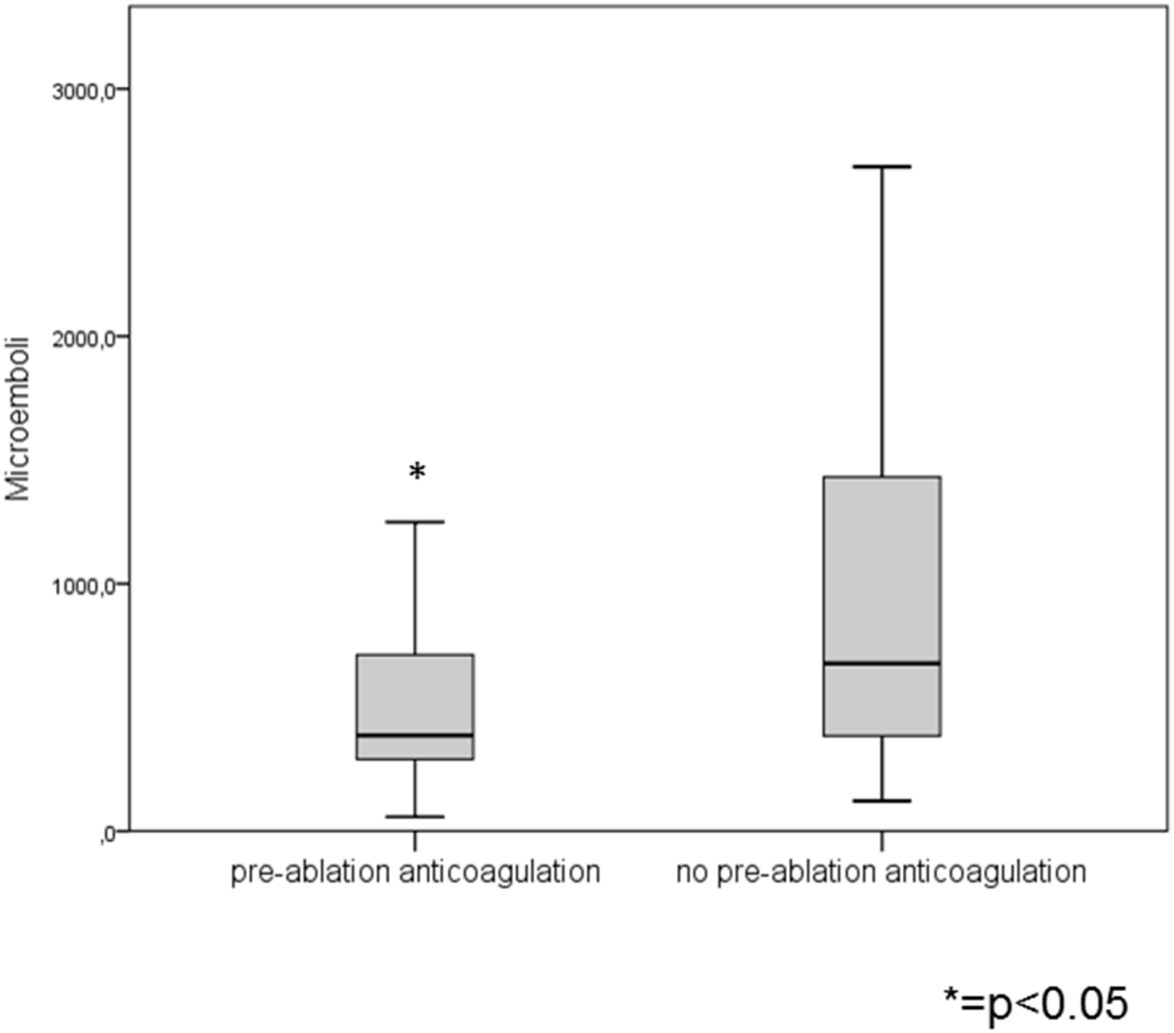
MES according to Phenprocoumon/Dabigatran vs. no anticoagulation Therapy pre-ablation. Anticoagulation with Phenprocoumon or new anticoagulant drugs vs. no therapy resulted in significantly reduced MES-rates (387 (289, 747) vs. 678 (378, 1447); p = 0.005).

**Fig 2:**
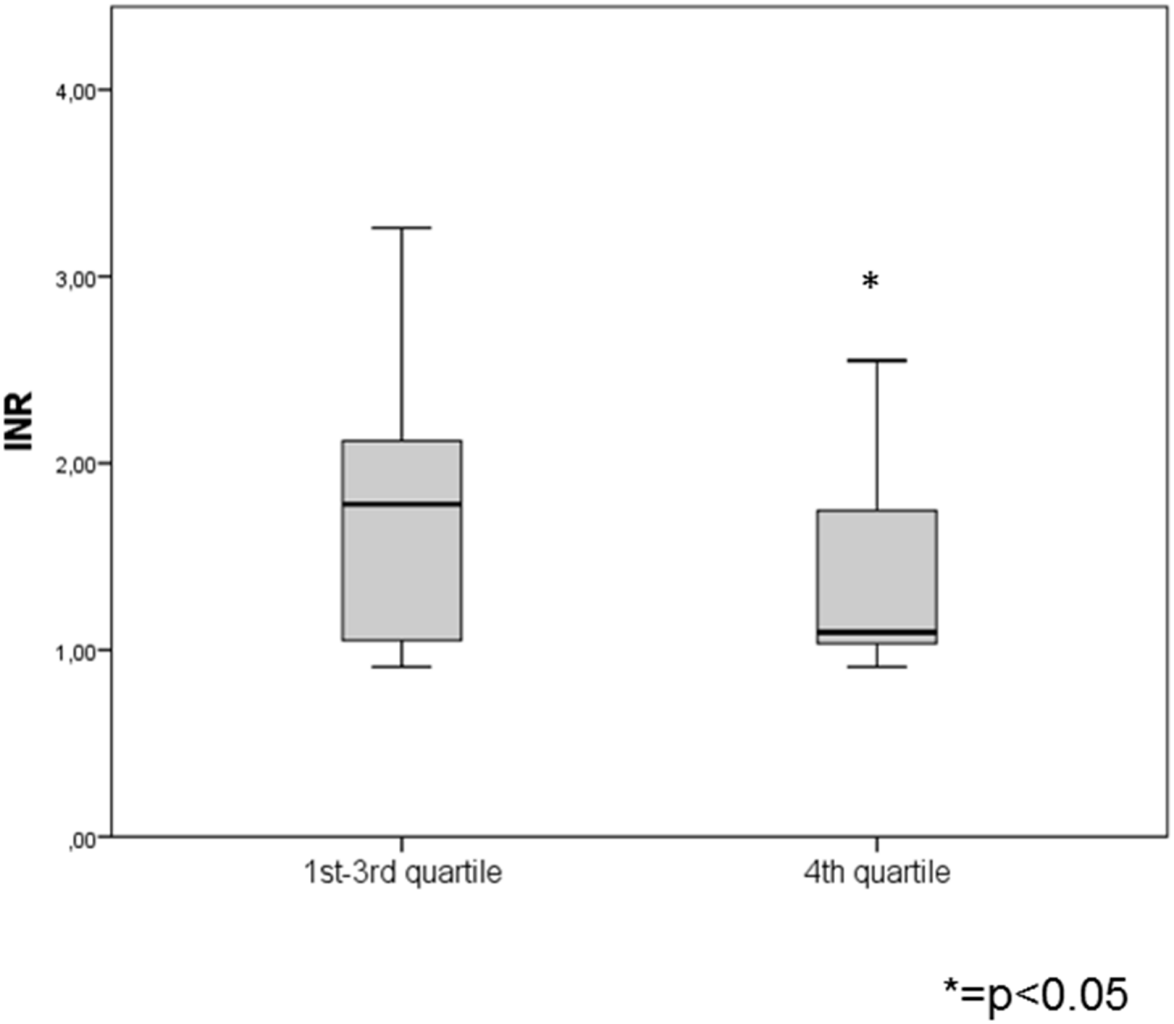
INR values within the first three and the highest quartile of MES counts. INR values in patients within the first three quartiles were significantly higher than in the 4^th^ quartile (1.78 (1.05, 1.8) vs. 1.09 (1.03, 1.78); p = 0.029).

A total number of 118 patients were included into the analysis. The baseline characteristics are shown in Table 1. Patients with pre-ablation anticoagulation (63 patients with Phenprocoumon and 6 patients with Dabigatran) had significantly less MES than patients without pre-ablation anticoagulation (387 (289, 747) vs. 678 (378, 1447); p = 0.005) (Fig 1) and were significantly more often found within the 1^st^-3^rd^ quartile of the overall MES count than in the 4^th^ quartile (65.6% vs. 35.7%; p = 0.005). Additionally, INR values in patients within the first three quartiles were significantly higher than in the 4^th^ quartile (1.78 (1.05, 1.8) vs. 1.09 (1.03, 1.78); p = 0.029) (Fig 2). All other baseline characteristics as well as the calculated scores were not significantly different between patients with a lower (1^st^-3^rd^ quartile) or higher (4^th^ quartile) MES count.

**Table 1:** Baseline characteristics.

	All	1-3^rd^ Quartile	4^th^ Quartile	p
Total, n (%)	118 (100.0)	90	28	
Age [years]	59.5 (±12.0)	60.3 (±11.5)	56.9 (±13.1)	0.160
Male	85 (72.0)	68 (75.6)	17 (60.7)	0.126
Female	33 (28.0)	22 (24.4)	11 (39.3)	
Hypertension	77 (65.3%)	60 (66.7%)	17(60.7%)	0.563
Diabetes	6 (5.1%)	6 (6.7%)	0	0.161
Heart failure	26 (22%)	22 (24.4%)	4 (14.3%)	0.257
LV Ejection Fraction [%]	60	60	60	0.854
	55, 60	54.5, 62.25	55.75, 60	
LA-diameter [mm]	39.5 (±6.6)	40.4 (±5.9)	36.6 (±8.1)	0.051
Body Mass Index (BMI)	27.5	27.5	27.4	0.42
	25.5, 30.2	25.5, 30.4	24.6, 29.1	
NYHA-class	1	1	1.5	0.79
	1, 2	1, 2	1, 2.75	
EHRA-class	2	2	2	0.3
	2, 3	2, 3	2, 3	
Charlson Comorbidity Index	0.5	1	0	0.19
	0, 2	0, 2	0, 2	
CHA_2_DS_2_VaSc-Score	2	2	1	0.36
	1, 3	1, 3	0.25, 3	
Antiarrhythmic Drug Therapy	101 (85.6%)	74 (82.2%)	27 (96.4%)	0.062
Diuretics	21 (17.8%)	18 (20.0%)	3 (10.7%)	0.262
ACE-/AT1-Antagonists	44 (37.3%)	33 (36.7%)	11 (39.3%)	0.802
Any Anticoagulation	69 (58.5%)	59 (65.6%)	10 (35.7%)	0.005
No Anticoagulation	49 (41.5%)	31 (34.4%)	18 (64.3)	0.005
NOAC	6 (5.1%)	6 (6.7%)	0	0.16
INR	1.58	1.78	1.09	0.029
	1.05, 2.09	1.05, 1.8	1.03, 1.78	
Heart rate [1/min]	65	69	60.5	0.19
	54.75, 80	55, 84.25	53.25, 76.75	
SR at admission	80 (67.8%)	57 (63.3%)	23 (82.1%)	0.063

Baseline characteristics: Pre-ablation OAC and increased INR values correlate with decreased MES-rates.

### Procedural characteristics

The analysis of the procedural characteristics is shown in Table 2. As previously described[3] the ablation approach had a profound influence on MES rates. Patients in the PVAC-all group were at the highest risk to have higher MES counts (within the 4^th^ quartile) when compared to patients in the iRF- and PVAC-red-groups (60.7 vs. 25.0% vs. 14.3% respectively (p<0.001).

**Table 2:** Procedural characteristics.

	All	1-3^rd^ Quartile	4^th^ Quartile	P
pRF	69 (58.5%)	62 (68.9%)	7 (25.0%)	<0.001
PVAC-red (red electrodes)	20 (16.9%)	16 (17.8%)	4 (14.3%)	
PVAC-all (all electrodes)	29 (24.6%)	12 (13.3%)	17 (60.7%)	
Procedure time [min]	166	173.5	152.5	0.129
	133.8, 195.8	135, 205	113.8, 189.5	
Fluoroscopic time [min]	23.8	24.2	21.79	0.092
	16.3, 30.1	17, 31	15.2, 26.3	
RF-energy time [min]	29.5	38	22.5	0.012
	19, 50	19.78, 51.25	18, 29.75	
Normal Anatomy	80 (67.8%)	61 (71.8%)	19 (70.4%)	0.889
Anatomical PV Variant*	32 (27.1%)	24 (28.2%)	8 (29.6%)	
No CT performed	6 (5.1%)			
Cardioversion in EP Study	39 (33.1)	29 (32.2)	10 (35.7)	0.732
ACT value [sec]	239 (213, 266)	233 (196, 275)	242 (220, 261)	0.38

* = Anatomical variants analyzed included: left common outflow (LCO), left common trunk (LCT), right common outflow (RCO), right middle pulmonary vein (RMPV), right top pulmonary vein (RTPV), early branching (EB) as previously described[5]. RF-energy delivery (OR 0.97 (0.94–0.99), p = 0.009) is a predictor of MES counts within the 4th quartile using univariable analysis.

Patients with a pulmonary vein variant were not at a higher risk for higher MES counts. A total of 2 patients developed a potentially thromboembolic event which was possibly associated with the PVI. One patient in the phased RF group developed transient, asymptomatic ST-segment-elevation in leads II, III and aVF during ablation and one patient suffered from a transient ischemic event after the first ablation impulse. Another patient in the irrigated RF group developed a pericardial effusion that required external drainage.

### Predictors for MES occurrence

Univariable analysis showed that the ablation approach (p<0.001), a pre-ablation anticoagulation (OR 0.29 (0.12–0.72), p = 0.007) and the amount of RF-energy delivery (OR 0.97 (0.94–0.99), p = 0.009) to be the strongest predictors of MES counts within the 4^th^ quartile.

When these parameters were included in a multivariable model the ablation approach stayed the strongest predictor (p<0.001), while the amount of RF-energy delivered was no independent predictor of higher MES counts (OR 0.99 (0.96–1.03), p = 0.69). However, a pre-ablation anticoagulation stayed an independent predictor of a MES-count within the 4^th^ quartile (OR 0.3 (0.11–0.82), p = 0.019).

## Discussion

MES are frequently observed in PVI. Several studies have been able to strengthen the role of MES as a surrogate parameter for thromboembolic activity in patients undergoing PVI[3, 4, 7]. However, patient-specific parameters and predictors of cerebral microemboli have not been tested for their possible association with thromboembolic activity during atrial fibrillation ablation.

Our findings suggest that clinical predictors of thromboembolism in AF patients do not correlate with intraprocedural MES-Rates during PVI. Especially the CHA_2_DS_2_-VASc-Score or its individual parameters did not predict higher MES-rates. Other clinical parameters which might suggest a patient-specific origin of MES e.g. other concomitant diseases such as structural heart disease of any cause or decreased left ventricular ejection fraction did not go along with significantly altered MES-rates. Interestingly, also anatomical features which might hinder the ablation procedure such as a dilatation of the left atrium or pulmonary vein variants did not accompany different MES-rates. This suggests that the formation of embolic signals during PVI might rather be a technical then a patient-specific, pathophysiological problem (Tables 1 and 2). This hypothesis is supported by other groups which have been able to reduce the number of MES during atrial fibrillation ablation with technical modifications and the use of newer generator systems[7]. In accordance with this hypothesis we were able to confirm the correlation of ablation type with decreased MES-rates as previously described[3]. Specifically, we were able to confirm that the use of a fully activated PVAC^®^ system as compared to iRF and PVAC-red leads to increased MES-formation.

Additionally, we were able to detect a correlation of pre-ablation anticoagulation with either Phenprocoumon or Dabigatran and significantly decreased MES-rates (p = 0.005). In correspondence to this we also observed that higher INR values on the day before PVI were significantly higher among patients within the first three quartiles of the total MES count. Finally our multivariable logistic regression analysis revealed that pre-ablation anticoagulation is an independent predictor to be within the three quartiles with lower MES counts. Although Phenprocoumon was paused 24h prior to PVI and INR values on the day of the ablation were just below 2.0, the remaining anticoagulating effect of Phenprocoumon during PVI was potentially reduced but sufficient to significantly influence MES-rates. Dabigatran was paused at least 24h prior to the procedure so a reduced anticoagulating effect with patients in this group was also potentially evident, however we only had 6 patients receiving this drug. These results correspond to data from other investigators, which were able to detect a decreased number of thromboembolic events after PVI while using a combined continuous periprocedural anticoagulation regime of low molecular weight heparin and ASS in patients with low thromboembolic risk[8]. These results are also in accordance with the COMPARE trial where performing catheter ablation of AF without warfarin discontinuation reduced the occurrence of periprocedural stroke and minor bleeding complications compared with bridging with low-molecular-weight heparin[9]. We show that pre-ablation anticoagulation is directly associated with a decreased MES-rate during PVI. We were not able to detect a correlation of MES- with ACT-values.

Currently it is unclear, whether a prophylactic anticoagulation before the PVI procedure is recommendable for patients with a CHA_2_DS_2_-Vasc-Score of 0, paroxysmal atrial fibrillation and sinusrhythmn[10]. Other observational studies previously supported an approach of performing ablation with a therapeutic INR, which may reduce the risk of ablation-related thromboembolism in low risk patients[11]. Based upon our data we conclude that a preexisting anticoagulation with either Phenprocoumon or Dabigatran might help to decrease the formation of MES and hence might reduce the risk of thromboembolic events. However, our study cannot detect the impact of MES-signals on cerebral anatomy since we could not correlate our data with routine MRI-scans.

### Conclusions

Although pre-procedural anticoagulation status and PVI ablation approach is independently predictive for MES formation CHA_2_DS_2_VASc-Score or other patient specific clinical parameters seem to have no impact on embolic burden during AF ablation. Therefore, it might be beneficial to start anticoagulation before the AF-ablation procedure.

### Limitations

Our retrospective approach cannot predict whether these conclusions are valid in a prospective analysis. In addition we performed a single center study which therefore bares the risk of an investigator-specific bias with regard to the PVAC^®^-procedure and the MES detection. We also did not specifically monitor the quality of wall contact during PVI by e.g. contact force measurements. Also, this study was not designed to test the effect of a continuous anticoagulation regime during PVI vs. a placebo group. Therefore, the conclusions drawn from this retrospective approach have to be further validated in a prospective, placebo-controlled clinical study. We also did not correlate the MES detection with routine MRI scans after the procedure. The pulmonary vein isolations in this study were performed in an era where we paused OAC prior to the intervention.

## Supporting Information

S1 Table Individual patient data. The results from the statistical analysis are based on this data.
(XLSX)
